# The World’s Columbian Dental Congress

**Published:** 1892-09

**Authors:** 


### THE WORLD'S COLUMBIAN DENTAL CONGRESS.

The anticipation that this Congress would be fully organized at Niagara Falls was not fulfilled, as the election of the superior officers was postponed until October.

It was with some regret that a disposition was noticed to urge certain men for the highest position. There should be but one rule for the government of the appointing power, and that should be peculiar fitness. We have avoided this subject in the past, as it was felt that the committee having it in charge was competent to meet all difficulties. It was clearly apparent, however, at Niagara that the spirit of politics was working a baleful influence in this as it has in the past in other organizations, and unless positively met might result in serious injury.

It seems to us that but three considerations should claim the attention of the appointing power. The person suggested for the highest place should be one who has been in constant touch with the development of dentistry in America for the past half-century. He should be well versed in parliamentary usage, and be familiar with men in general and the locality of the meeting in particular. If these qualifications be accepted, the circle of choice will necessarily be a narrow one. It would seem to be very inappropriate to place a comparatively young man at the head of a meeting organized to represent to the dentists of the world the progress made during the present century. The past is best represented by age; progress by youth.

The committees having the work of the Congress have accomplished their arduous labor very satisfactorily, and we entertain no misgivings as to the final result.

